# Awareness and Use of Saw Palmetto and Other Alternative Medicines Used for Treating Prostate Diseases Among Men Visiting a Urology Clinic in Guyana

**DOI:** 10.7759/cureus.115315

**Published:** 2026-08-27

**Authors:** Akesha P Murray, Rajendra Sukhraj

**Affiliations:** 1 Department of Surgery - Urology Section, Georgetown Public Hospital Corporation, Georgetown, GUY

**Keywords:** alternative medicine, benign prostatic hyperplasia, guyana, herbal medicine use, lower urinary tract symptoms, prostate disease, saw palmetto

## Abstract

Introduction: Herbal medicine remains widely used in the Caribbean, including Guyana, where traditional remedies are often used alongside conventional medical care. Saw palmetto (*Serenoa repens*) is internationally recognized as a common herbal supplement for benign prostatic hyperplasia (BPH) and lower urinary tract symptoms, but local data on its use among Guyanese men with prostate diseases are limited. The objective of this study is to assess awareness of saw palmetto and estimate the prevalence of saw palmetto and other herbal medicine use among men with prostate diseases attending the urology clinic at Georgetown Public Hospital Corporation, Guyana, and to describe the perceived effectiveness, concurrent use with prescribed medications, and disclosure to healthcare providers.

Method*:* A prospective cross-sectional study was conducted at the Urology Section, Department of Surgery, Georgetown Public Hospital Corporation, Guyana, between July and September 2024. Male patients aged 40 years and older with a documented diagnosis of BPH, prostatomegaly, or prostate cancer were recruited using convenience sampling. A structured, interviewer-administered questionnaire was used to collect data on demographics, medical history, saw palmetto awareness and use, other herbal medicine use, treatment beliefs, concurrent use with prescribed medications, perceived effectiveness, and disclosure to healthcare providers. Data were analyzed using descriptive statistics.

Results*:* A total of 200 patients were enrolled in this study. Of the 200 participants, 88 (44%) were aged 70 years and above, 81 (40.5%) were aged 60-69 years, 29 (14.5%) were aged 50-59 years, and two (1%) were aged 40-49 years. The region of residence was reported by all 200 participants, of whom 146 (73%) reported being from region 4. The diagnoses documented in the clinic charts included BPH in 88/200 (44%), prostatomegaly in 86/200 (43%), and prostate cancer in 26/200 (13%). Among the 189 participants who responded to the saw palmetto use question, 31/189 (16.4%) reported use for prostate health. Separately, 84/200 (42.0%) reported using other herbal products for prostate health. Among the 38 participants who responded to the multiple-response question on sources of information for saw palmetto, 23/38 (60.5%) selected friends/family, 6/38 (15.8%) selected the internet, 4/38 (10.5%) selected a physician, and 3/38 (7.9%) selected a pharmacist. Of the 185 participants who responded to the perceived-effectiveness question, 157/185 (84.9%) were unsure, 24/185 (13.0%) believed it was effective, and 4/185 (2.2%) considered it ineffective. Of the 200 participants, 28/200 (14.0%) reported discussing herbal product use with their healthcare provider, whereas 172/200 (86.0%) reported not disclosing this information.

Conclusion*:* Saw palmetto awareness and use were relatively low among the men with prostate diseases attending the urology clinic at the Georgetown Public Hospital Corporation, Guyana, despite frequent use of other herbal and alternative medicines. Participants appeared to rely more on locally available traditional remedies, particularly nettle root, rather than on internationally marketed prostate supplements. The high rate of non-disclosure highlights the need for clinicians to routinely ask about herbal medicine use, provide evidence-based counseling, and address potential risks, including herb-drug interactions.

## Introduction

Prostate diseases, including benign prostatic hyperplasia (BPH) and prostate cancer, are important causes of morbidity among older men worldwide. In countries such as Guyana, where traditional remedies remain widely used and access to healthcare may vary, men with urological symptoms may seek herbal or complementary therapies in addition to conventional treatment. Saw palmetto (*Serenoa repens*) is among the most recognized herbal products used for prostate-related symptoms and has been the subject of considerable clinical investigation [1].

Saw palmetto extract was historically used for urinary symptoms, reproductive health, and libido. Its modern use is mainly directed toward BPH and associated lower urinary tract symptoms (LUTS) [2]. Proposed mechanisms include androgen receptor modulation, anti-inflammatory activity, inhibition of cellular proliferation, and partial inhibition of 5α-reductase, the enzyme that converts testosterone to dihydrotestosterone [3,4]. Despite these proposed biological effects, clinical findings remain inconsistent, with some studies suggesting symptomatic benefit and others showing no significant improvement compared with placebo, while systematic reviews similarly questioned the magnitude of benefits associated with saw palmetto [5,6]. In contrast, more recent systematic reviews of *Serenoa repens*, including specific preparations such as the hexanic lipidosterolic extract, have reported improvements in some LUTS-related outcomes [7,8].

In the​‍​‌‍​‍‌​‍​‌‍​‍‌ Caribbean, particularly Guyana, the use of herbal medicines has been around for a long time. This usage has been heavily influenced by people's culture, their economy, and how they can get medicines. Though the use of herbal products for the health of the prostate is quite popular, little has been done to find out the usage, prevalence, and perceived effectiveness of such treatments among men with prostate diseases in Guyana. Doctors need to understand patients' habits because it is important for patient counselling and communication regarding concurrent use with prescribed medications.

A previous study by Persaud et al. examined the knowledge and prevalence of saw palmetto and other herbal product use among men with prostate disease in Trinidad and Tobago [9]. However, comparable data among men attending urology services in Guyana remain limited. The present study was developed to examine these issues within the Guyanese clinical setting.

Georgetown Public Hospital Corporation (GPHC) serves as the main referral hospital in Guyana. The urology section of the surgery department has noted a significant prevalence of herbal supplement use among patients, especially those with prostate diseases. This study aims to address the lack of information regarding the awareness and usage of saw palmetto and other herbal remedies among men with prostate conditions visiting the urology clinic at GPHC.

The primary objective of this study was to estimate how prevalent the use of saw palmetto and other herbal medicines is among men with prostate diseases attending the clinic during the study period. The secondary objectives included assessing awareness of saw palmetto, identifying sources of information, evaluating perceived effectiveness, examining the concurrent use of these supplements with prescribed medications, and determining whether patients disclose their use of herbal medicines to healthcare providers. This research will provide valuable insights for healthcare professionals and guide future studies in this population.

## Materials and methods

We conducted a prospective cross-sectional study at the Urology Section, Department of Surgery, GPHC, between July and September 2024. Male patients aged 40 years and older, of all ethnicities and geographic locations, with a documented diagnosis of BPH, prostatomegaly, or prostate cancer, and attending the urology clinic at GPHC were eligible. The diagnoses for patients were confirmed by reviewing their clinic charts, imaging studies, and histopathology reports, where applicable. The diagnostic categories corresponded to those recorded in the clinical records; no additional study-specific adjudication criteria were utilized. Patients with self-diagnosed prostate diseases without a documented clinical diagnosis, cognitive impairment, or unwillingness to participate were excluded.

A convenience sampling technique was adopted. There was no specific sample size calculation. The sample size was set according to the number that seemed realistic to obtain within the research period. There are no diagnoses recorded in the clinic database at the urology clinic at GPHC; hence, the total number of patients with the relevant prostate diagnoses visiting the clinic could not be ascertained.

A total of 240 patients were approached during this study; however, 40 declined consent, leaving us with 200 who provided written consent and were enrolled, corresponding to a participation rate of 83.3%. Participants completed a structured, interviewer-administered questionnaire, as illustrated in the Appendices. Data collected included demographic characteristics, medical history, awareness and use of saw palmetto, use of other herbal medicines, perceived effectiveness, sources of information, treatment beliefs, concurrent use with prescribed medications, and disclosure to healthcare providers.

The questionnaire, as illustrated in the Appendices, was conducted in English by various interviewers from the urology department at GPHC. The work of Persaud et al. (2017) provided the theoretical basis for the research, yet the questionnaire created by them was neither reproduced nor adapted in any way [9]. The questionnaire used in the research was created by the authors themselves using the results of the aforementioned study and was developed specifically for the aims of this study and its Guyanese context. It was neither validated nor pilot-tested and served the purpose of assessing awareness and use of the intervention.

Use of saw palmetto in this study was defined as a participant self-reporting that they had used saw palmetto for prostate health. Use of other herbal medicines in this study was defined as self-reporting the use of herbal medicines other than saw palmetto for prostate health. Concurrent use was defined as self-reporting the use of herbal medicines along with the prescription medicine as advised by the participant’s doctor. No specific time period for recalling herbal use was mentioned in the survey.

The data were entered in Microsoft Excel (Microsoft Corporation, Redmond, WA) and analyzed using IBM SPSS software, version 30 (IBM Corp., Armonk, NY). Categorical variables were described using descriptive statistics by frequency and percentage. Since not all study participants had responded to all questionnaire questions, percentages were based on the number of valid responses to that particular question, and where necessary, denominators were specified at the individual question level. Unfilled or partially filled responses were not included in the denominator for the particular question only. Responses marked "unsure" were included as one of the response options. For multiple-response questions, each response option was considered independently, and percentages were obtained using the number of valid respondents to that particular question; thus, the total of these percentages may be greater than 100%.

Ethical approval was obtained from the Institutional Review Board of the Ministry of Health and the Research Committee at Georgetown Public Hospital Corporation, Guyana. Written informed consent was obtained from all participants.

## Results

Demographic characteristics

A total of 200 male patients were included in the study. Among these, 88/200 (44.0%) were aged 70 years and above, 81/200 (40.5%) were aged 60-69 years, 29/200 (14.5%) were aged 50-59 years, and 2/200 (1.0%) were aged 40-49 years, as illustrated in Table 1. Ethnicity was recorded for 199 participants, with 132/199 (66.3%) participants identified as African, 39/199 (19.6%) as East Indian, 21/199 (10.6%) as mixed, and 7/199 (3.5%) as Amerindian, as presented in Table 1.

**Table 1:** Demographic characteristics of study participants.

Variable	Frequency (n)	Percent (%)
Age (N = 200)		
>70	88	44
60-69	81	40.5
50-59	29	14.5
40-49	2	1
Gender (N = 200)		
Female	0	0
Male	200	100
Ethnicity (N = 199)		
African	132	66.3
East-Indian	39	19.6
Mixed	21	10.6
Amerindian	7	3.5
Educational level (N = 200)		
Primary education	116	58
Secondary education	68	34
Tertiary education	16	8
Occupation (N = 200)		
Retired	112	56
Employed	46	23
Unemployed	40	20
Other	2	1

Educational level was recorded for all 200 participants. Primary education was the highest level of education for 116/200 (58%) of participants, while 68/200 (34%) had secondary education and 16/200 (8%) had tertiary education, as seen in Table 1. Occupation was also recorded for all 200 participants, with 112/200 (56%) retired, followed by 46/200 (23%) employed and 40/200 (20%) unemployed, and 2/200 (1.0%) reported another occupational status, as seen in Table 1. While participants came from eight of Guyana's 10 administrative regions, 146/200 (73%) resided in region 4, which accounted for the majority.

Diagnosis

Participants had established prostate-related diagnoses documented in the clinic charts, with BPH in 88/200 (44%), prostatomegaly in 86/200 (43%), and prostate cancer in 26/200 (13%). The most frequently reported duration of disease was one to three years, accounting for 88/200 (44%) respondents.

Awareness and use of saw palmetto

Awareness of saw palmetto was low, with 43/200 participants (21.5%) reporting that they had heard of it. Among the 189 participants who responded to the question regarding saw palmetto use, 31/189 (16.4%) reported having used it for prostate health. Among the 38 participants who responded to the multiple-response question on sources of information, 23/38 (60.5%) selected friends/family, 6/38 (15.8%) selected the internet, 4/38 (10.5%) selected a physician, and 3/38 (7.9%) selected a pharmacist. Given the multiple responses permitted, the percentages may sum to more than 100%. Among the 31 participants who responded to the frequency question, 26/31 (83.9%) reported using saw palmetto daily. Of the 41 participants who responded to the adverse-effects question, 1/41 (2.4%) reported an adverse effect, described as constipation.

Of the 185 participants who responded to the perceived-effectiveness question, 157/185 (84.9%) were unsure whether saw palmetto was beneficial, 24/185 (13.0%) believed it was effective in improving their prostate condition, and 4/185 (2.2%) considered it ineffective. Among the 37 people who answered the question regarding their perception of the benefits associated with saw palmetto, the most common one identified was improved urinary flow in 18/37 (48.6%), followed by reduced prostate size in 6/37 (16.2%), reduction of inflammation in 3/37 (8.1%), prevention of prostate cancer in 3/37 (8.1%), reduced urinary frequency in 2/37 (5.4%), and reduced hesitancy in 1/37 (2.7%). Of the 37 who responded, six participants did not select a perceived benefit but instead reported never having used it. Multiple answers were allowed; thus, the percentages may not add up to 100%.

Use of other herbal medicines

Among 200 participants, 84/200 (42%) reported the use of herbal medicines other than saw palmetto. Among the 86 participants who provided a response to the multiple-response question on specific herbal products, 66/86 (76.7%) reported nettle root use, often in combination with other products such as dandelion, pumpkin seed, and soursop leaf. A total of 194 participants responded to whether herbal products were a better alternative to conventional medicine, with 15/194 (7.7%) answering yes. Meanwhile, 196 participants responded to concurrent use, with 47/196 (24.0%) reporting using herbal products alongside physician-prescribed medications. Among all 200 participants, 28/200 (14.0%) reported discussing herbal product use with their healthcare provider, whereas 172/200 (86.0%) reported that they did not. Commercial prostate supplements such as ProstaGenix, Super Beta Prostate, selenium, and lycopene were not used by any participants surveyed.

## Discussion

The findings in this study provide valuable initial insights into the awareness and prevalence of use of saw palmetto and other herbal medicines used to treat prostate diseases among men with prostate diseases attending the urology clinic at GPHC in Guyana. It is the first study of its kind conducted in Guyana. Several key themes emerge that both align with and diverge from existing regional and global literature.

Although saw palmetto is widely marketed internationally for LUTS and BPH, awareness and use were relatively low among men visiting the urology clinic at the GPHC. Of the 200 participants, only 43 (21.5%) were aware of saw palmetto, and 31 of 189 respondents (16.4%) reported that they used it. Additionally, 84 of 200 (42%) participants reported using other herbal medicines. This differs from findings by Persaud et al. (2017) in Trinidad and Tobago, where it was reported that herbal supplement use was among 42.5% of men, with saw palmetto used by 62.3% of supplement users [9]. These findings revealed that despite geographic and cultural similarities between these two Caribbean countries, herbal medicine practices and saw palmetto awareness and use differ between patients attending respective urology clinics.

The findings also suggest that Guyanese men may rely more heavily on locally available “bush medicine” than on internationally marketed prostate supplements. Nettle root was the most common herbal product reported and was frequently combined with dandelion, pumpkin seed, and soursop leaf. This pattern is consistent with Caribbean traditions in which natural products are often selected based on cultural familiarity, community knowledge, and local availability. A Jamaican study of prostate cancer patients similarly reported substantial use of herbal products, although guinea hen weed was the most commonly used remedy in that population [10].

Out of the total number of 185 respondents who answered the perceived effectiveness of saw palmetto question, 157 respondents (84.9%) were unsure about the effectiveness of saw palmetto, while 24 respondents (13.0%) considered saw palmetto to be effective. This is what the respondents thought about the effectiveness of saw palmetto and should not be interpreted as evidence of the clinical effectiveness of saw palmetto. This distinction is important because clinical studies evaluating saw palmetto have produced inconsistent findings. A randomized controlled trial and systematic review found no clear improvement in LUTS among men taking saw palmetto compared with placebo [5,6]. In contrast, other systematic studies and randomized controlled trials found that *Serenoa repens* improved the International Prostate Symptom Score and quality of life in men with BPH [7,8]. A systematic review and meta-analysis found that *Serenoa repens* was equally effective as tamsulosin in treating BPH [11]. This conclusion was based on parameters such as the International Prostate Symptom Score, quality of life, and post-void residual volume after at least six months of treatment.

One respondent reported constipation as an adverse effect. It is necessary to state that this is a self-reported adverse effect and not a causative relation of saw palmetto with constipation, as the present study did not evaluate the clinical causality or objectively measure the clinical outcomes associated with saw palmetto use.

When we looked at the 38 people who answered the question about where they obtained saw palmetto information, the numbers were quite telling. About 23 out of 38 participants (60.5%) said they talk to friends or family, six out of 38 participants (15.8%) reported using the internet to find saw palmetto information, four out of 38 participants (10.5%) spoke to a doctor, and three out of 38 (7.9%) asked a pharmacist. These results showed us that people often rely on sources to learn things, and some of these sources can be unreliable. It also shows that we really need discussions between patients and healthcare providers about herbal products. Looking at all 200 participants, only 28 out of 200 (14.0%) actually talked with their healthcare provider about herbal product use. The other 172, out of 200 (86.0%), did not talk to a healthcare provider.

Although this was a single-institution study, GPHC is Guyana’s main tertiary referral hospital and serves patients from across the country. The inclusion of participants from eight of the 10 administrative regions supports the relevance of these findings to the wider Guyanese population. However, this geographic diversity does not establish national representativeness because participants were recruited using convenience sampling from a single tertiary referral clinic and may introduce selection bias. Other limitations in this study included self-reported answers, which may be susceptible to recall and reporting biases. Furthermore, there was no preset period for the use of the herbal products. Lastly, the cross-sectional study design is a study that takes place at a single point in time and cannot be used to assess change over time.

## Conclusions

This study found that men with prostate diseases attending the urology clinic at GPHC in Guyana commonly reported using herbal products other than saw palmetto. Among respondents, nettle root was the most frequently mentioned herbal product, often used in combination with dandelion, pumpkin seed, and soursop leaf. Awareness and use of saw palmetto were relatively low among participants, and most were uncertain about its effectiveness; disclosing their use of herbal products to healthcare providers was uncommon. These findings reflect the participants' reported awareness, use, and perceptions of herbal products but do not establish the clinical effectiveness of these treatments. No participants reported using herbal supplements like Super Beta Prostate, lycopene, ProstaGenix, or selenium. The results also highlight the importance of healthcare providers routinely inquiring about herbal medicine use, offering balanced counseling, and discussing potential risks, including interactions with prescribed therapies.

## Appendices

**Table 2:** Questionnaire used for data collection to assess the knowledge and prevalence of saw palmetto and other herbal products used for treating prostate diseases among Guyanese men.

Section	Question number	Question	Response Option 1	Response Option 2	Response Option 3	Response Option 4	Response Option 5	Response Option 6	Response Option 7	Response Option 8
Section 1: Sociodemographic Information	1	Age	40-49	50-59	60-69	70 and above	_	_	_	_
	2	Sex	Male	_	_	_	_	_	_	_
	3	Ethnicity	African	East Indian	Amerindian	Chinese	Portuguese	Mixed	_	_
	4	Area/Region of residence								
	5	Educational level	No formal education	Primary education	Secondary education	Tertiary education	_	_	_	_
	6	Occupation	Employed	Unemployed	Retired	Other (please specify)	_	_	_	_
Section 2: Medical History	7	Do you have a diagnosis of prostate disease?	Yes	No	_	_	_	_	_	_
	8	If yes, what type of prostate disease?	Benign prostatic hyperplasia (BPH)	Prostate cancer	Prostatomegaly	_	_	_	_	_
	9	How long have you had this condition?	Less than 1 year	1-3 years	4-6 years	More than 6 years	_	_	_	_
Section 3: Use of Herbal Products	10	Have you heard of saw palmetto?	Yes	No	_	_	_	_	_	_
	11	Have you used saw palmetto for prostate health?	Yes	No	_	_	_	_	_	_
	12	If yes, how did you learn about saw palmetto?	Physician	Pharmacist	Friends/Family	Internet	Media (TV, Radio, Magazine)	Other (please specify)	_	_
	13	How frequently do you use saw palmetto?	Daily	Weekly	Monthly	Occasionally	_	_	_	_
	14	Have you experienced any side effects from using saw palmetto?	Yes	No	_	_	_	_	_	_
	15	If yes, please describe the side effects.								
Section 4: Knowledge and Beliefs	16	Do you believe that saw palmetto is effective in treating prostate diseases?	Yes	No	Unsure	_	_	_	_	_
	17	What benefits do you associate with saw palmetto?	Reducing prostate size	Improving urinary flow	Reducing inflammation	Preventing prostate cancer	Other (please specify)	_	_	_
	18	Do you use any other herbal products for prostate health?	Yes	No	_	_	_	_	_	_
	19	If yes, which one?	Nettle root	Selenium	Lycopene	Pumpkin seed	Soursop leave	ProstaGenix	Super Beta Prostate	Other (please specify)
	20	Do you believe that herbal products are a better alternative to conventional medicine for prostate diseases?	Yes	No	Unsure	_	_	_	_	_
	21	If yes, why?								
	22	Do you use herbal products along with your physician's prescribed medication(s)?	Yes	No	_	_	_	_	_	_
	23	If yes, why?								
Section 5: Health-Seeking Behavior	24	How often do you visit your healthcare provider for prostate health?	Every 6 months	Annually	Only when symptoms worsen	Rarely/Never	_	_	_	_
	25	Do you discuss the use of herbal products with your healthcare provider?	Yes	No	_	_	_	_	_	_
	26	If no, why not?	Don’t think it's important	Fear of disapproval	Prefer self-management	Other (please specify)	_	_	_	_
Section 6: Demographic Correlations	27	Do you think education level affects knowledge of herbal products for prostate health?	Yes	No	Unsure	_	_	_	_	_
	28	Do you think the region of residence affects the availability of herbal products for prostate health?	Yes	No	Unsure	_	_	_	_	_
Section 7: Feedback and Suggestions	29	Do you have any suggestions for improving awareness and education about herbal products for prostate health?								
